# Integrating bacterial molecular genetics with chemical biology for renewed antibacterial drug discovery

**DOI:** 10.1042/BCJ20220062

**Published:** 2024-07-03

**Authors:** Susannah L. Parkhill, Eachan O. Johnson

**Affiliations:** 1 Systems Chemical Biology of Infection and Resistance Laboratory, The Francis Crick Institute, London, U.K.; 2 Faculty of Life Sciences, University College London, London, U.K.; 3 Department of Chemistry, Imperial College, London, U.K.; 4 Department of Chemistry, King's College London, London, U.K.

**Keywords:** antimicrobial resistance, antibacterial drugs, drug discovery, genetics, molecular biology

## Abstract

The application of dyes to understanding the aetiology of infection inspired antimicrobial chemotherapy and the first wave of antibacterial drugs. The second wave of antibacterial drug discovery was driven by rapid discovery of natural products, now making up 69% of current antibacterial drugs. But now with the most prevalent natural products already discovered, ∼10^7^ new soil-dwelling bacterial species must be screened to discover one new class of natural product. Therefore, instead of a third wave of antibacterial drug discovery, there is now a discovery bottleneck. Unlike natural products which are curated by billions of years of microbial antagonism, the vast synthetic chemical space still requires artificial curation through the therapeutics science of antibacterial drugs — a systematic understanding of how small molecules interact with bacterial physiology, effect desired phenotypes, and benefit the host. Bacterial molecular genetics can elucidate pathogen biology relevant to therapeutics development, but it can also be applied directly to understanding mechanisms and liabilities of new chemical agents with new mechanisms of action. Therefore, the next phase of antibacterial drug discovery could be enabled by integrating chemical expertise with systematic dissection of bacterial infection biology. Facing the ambitious endeavour to find new molecules from nature or new-to-nature which cure bacterial infections, the capabilities furnished by modern chemical biology and molecular genetics can be applied to prospecting for chemical modulators of new targets which circumvent prevalent resistance mechanisms.

## Introduction

**Figure 1.**
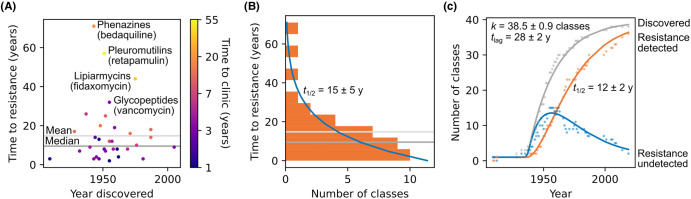
The rate of antibacterial drug discovery has reached a plateau, while the rate of emergence of new resistance is constant. (**A**) The time between discovery and resistance detection compared with the discovery year for classes of antibacterial drugs. Colour indicates the time between discovery and clinical use. Outliers in time to resistance (labelled with a canonical example in parentheses) are characterised by a longer gap between discovery and clinical use. (**B**) The distribution of time between discovery and resistance for classes of antibacterial drugs follows an exponential distribution (Poisson maximum likelihood fit shown by blue line), with a half-life of ∼15 years (95% confidence interval: 10–20 years). (**C**) Poisson maximum likelihood best fits for the dynamics of antibacterial drug discovery. The number of classes with resistance not-yet-detected (blue line) as a function of time was modelled as the difference between the number of discovered classes, *D* (grey line), and the number of classes with detected resistance, *R* (orange line). *R* was modelled as exponential growth with rate 1*/t*_1/2_. *D* was modelled as the coupon collector problem when sampling from *k* antibacterial drug classes at a constant rate *n* after a ∼28 year lag phase, *t*_lag_. Data were obtained from Stennett etal. [3].

Antibacterial drugs form the backbone of modern medicine by not only curing infections but also supporting essential interventions including organ transplantation and cancer treatment. Alarmingly, in 2019, an estimated 4.95 million deaths worldwide were associated with antimicrobial resistance [1,2]. Compounding this issue, the half-life of an antibacterial agent in the clinic before detection of resistance is ∼15 years (Figure 1A,B), requiring constant discovery to sustain an inventory of clinically useful antibacterial drugs which circumvent prevalent resistance mechanisms. In the last 30 years, the rate of discovery of these drugs has fallen behind the emergence of new resistance, such that there is now resistance to every clinically approved antibiotic (Figure 1C). As a result, antibacterial drugs are a perishable common good requiring a strategy for sustainable discovery.

The present torpor emerges from peculiarities of the history and practice of antibacterial drug discovery. First, the failure of target-based discovery [4,5] has made the target-agnostic phenotypic approaches necessary, resulting in increasing re-discovery of easily drugged targets. Second, a focus on natural products has led to an underdeveloped antibacterial therapeutics science which would otherwise guide the discovery and development of new synthetic antibacterial drugs.

Examining the early history of antibacterial drugs reveals unexplored paths to renewed discovery which might guide the application of the powerful genome-scale genetic and chemical tools now at our disposal. In an early example of integrative chemical biology, in 1882, the dye methylene blue revealed to Robert Koch the bacterium *Mycobacterium tuberculosis* as the cause of tuberculosis, inspiring the development of his eponymous postulates. This fundamental biological discovery using chemistry inspired potential cures: contemporaneously with Koch, Paul Ehrlich's selective staining of specific mammalian cells, such as mast cells, led to concept of the magic bullet, a chemical which partitions into disease-causing cells and harms them, leaving host or normal cells intact. Soon after, trypan red (named for its activity against trypanosomes) was discovered as the first antimicrobial magic bullet in 1904. In 1909, Ehrlich himself identified, with the assistance of Sahachiro Hata, the first commercial antibacterial agent, arsphenamine, as a cure for syphilis. Inspired by this progress, dye companies pursued systematic derivatisation and screening of their inventories and reaction side-products against microbes [6,7]. This effort led to the first wave of discovery of effective antibacterial drugs starting with the prodrug, prontosil by Gerhard Domagk in 1932 and its active metabolite, sulfanilamide, in 1935.

The second wave of antibacterial drug discovery followed the isolation of penicillin from the *Penicillium chrysogenum* fungus in 1940 [8],demonstrating for the first time that natural products could be harnessed not only as narcotics (such as opium and digitoxin) but also as life-saving medicines. This conceptual revolution provided benefits beyond curing infectious disease, including cancer treatment (anthracyclines, etoposide, vinca alkaloids) and prevention of organ transplant rejection (rapamycin, cyclosporin). For most bacterial infections, natural product screening and development of semi-synthetic analogues overtook synthetic chemistry approaches in a golden era of antibiotic discovery during the mid-20th century [7,9,10]. Now, 69% of antibacterial drugs are natural products derived from soil microbes, in contrast with the entire pharmacopoeia which comprises 38% natural products [11].

Notable exceptions are the antituberculosis drugs, most of which are synthetics from early screening of dye manufacturing by-products, despite the first effective tuberculosis chemotherapy being streptomycin, a natural product derived from the bacterium *Streptomyces griseus*. Streptomycin, discovered by Albert Schatz, Elizabeth Bugie, and Selman Waksman in 1944, caused a high incidence of allergies and relatively fast resistance acquisition by *M. tuberculosis* (factors which led the death of the author George Orwell in 1950). Therefore, it was eventually combined with synthetic *para*-aminosalicylic acid as the standard of care following a landmark clinical trial in 1950 [12]. Since then, streptomycin is no longer routinely used, with rifampicin the only natural product among first-line antituberculosis drugs.

The post-genomic age of the late 20th century promised a third wave of antibacterial drug discovery. It was thought that molecular biology, which provided the ability to identify new targets unique to pathogens, would yield numerous magic bullets through rational design [13]. However, high-throughput biochemical screening against known or novel targets *in vitro* over the last two decades have resulted in no clinical antibacterial candidates, with retrospective analyses noting an apparent bias towards tight-binding compounds with high lipophilicity, and subsequent difficulties in developing whole-cell activity [4,14]. This failure of target-based screening taught the harsh lesson that a drug is more than an avid binder of a validated target.

As a result, target-agnostic phenotypic screening for antibacterial candidates has returned to prominence along with an appreciation for natural products. However, discovery is stymied by the coupon collector problem, where drawing from the same pool of natural products at first yields a rapid increase in new discoveries — the golden age — followed by repeated re-discovery (Figure 1C). This phenomenon became so acute that by 1958 commercial natural product mining efforts invested in dereplication platforms to weed out rediscoveries early in their pipeline [15], but now with the most prevalent agents already discovered, to yield one new antibacterial natural product requires screening around 10^7^ new bacterial species [16].

Therefore, instead of a third wave of antibacterial drug discovery, there is now a discovery bottleneck (Figure 1C). Since the staining of *M. tuberculosis* with methylene blue, our understanding of bacterial biology and infection processes has increased dramatically, but in the last 10 years, all 19 approved new antibacterial drugs belonged to existing classes, except for antituberculosis drugs pretomanid (2019) and delamanid (2014) [17–20]. Of seven antibiotics in new drug application or Phase III clinical trials at the end of 2022, only gepotidacin (for urinary tract infections and gonorrhoea) and zoliflodacin (for gonorrhoea) are novel pharmacophores and only epetraborole (for *Mycobacterium avium* infection) has a new mechanism of action. None are found in nature, indicating the potential of synthetics to furnish new drugs with new mechanisms of action.

The proportion of synthetic compounds (from 44% to 61%) and of novel pharmacophores or mechanisms of action (28% to 65%) has increased in all phases of clinical trials from 2011 to 2022, indicating a promising shift away from me-too drugs and natural products to which resistance likely already exists in the environment [17,21]. However, unlike natural products which are selected and enriched by billions of years of microbial antagonism [22], the vast synthetic chemical space requires artificial curation, which can be assisted by integrated chemical and molecular genetic approaches to discover new compounds active against pathogenic bacteria.

### Integrating chemistry with genetics for renewed discovery

The conventional role of molecular genetics in drug discovery has been to implicate gene products in disease, thus prioritising them for target-based compound screening. However, unlike many drugs such as ivacaftor (restoring CFTR activity) for cystic fibrosis, imatinib (inhibiting tyrosine kinase) for leukaemia, and saquinavir (protease inhibitor) for HIV, the targets of antibacterial drugs were rarely known before clinical use. Indeed, the mechanism is still debated in several cases, including, for example, pyrazinamide for tuberculosis [23,24]. This ignorance does not preclude clinical value, but rather indicates complex, multi-target activities which drive efficacy and raise the barrier to resistance. As a result, most approved antibacterial drugs were empirically identified as killing a pathogen or curing a host [5], with target-based efforts to discover new antibacterial drugs almost universally failing [4].

**Figure 2.**
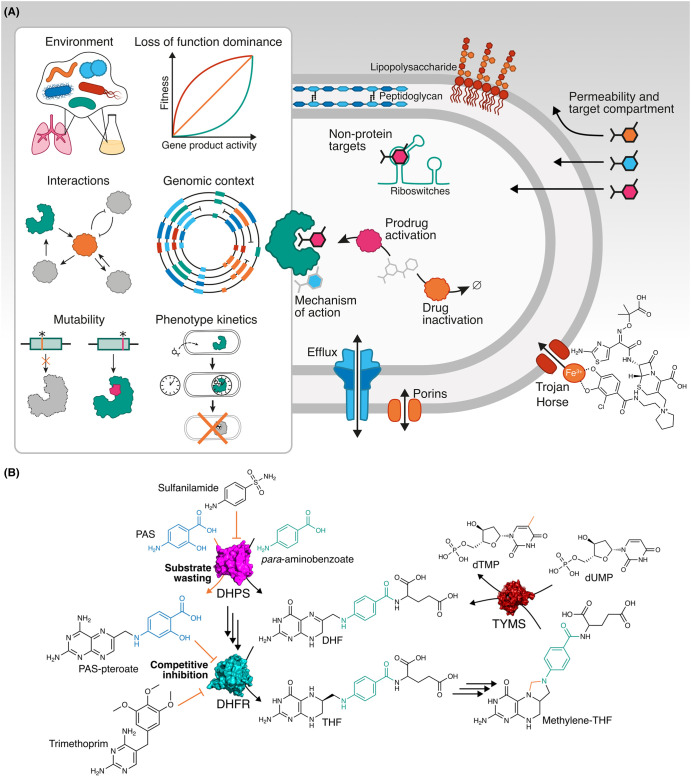
Biological approaches integrated with chemical understanding are essential for antibacterial drug discovery. (**A**) Putative targets of hit compounds can be prioritised using genetic approaches (left panel) to assess their likely efficacy and propensity for resistance. The fate and efficacy of bioactive compounds is not only determined by target, but also by chemical properties which influence uptake, mechanism of action, and, again, propensity for resistance. (**B**) The folate biosynthesis pathway is an exemplar of multiple mechanisms of action including competitive inhibition (trimethoprim) and substrate wasting (PAS), as well as how knowledge of biological networks can explain synergy (trimethoprim and sulfonamides) and resistance liabilities (TYMS loss of function renders DHFR non-essential). Atoms conserved in downstream metabolites are indicated in colours. DHFR, dihydrofolate reductase; DHF, dihydrofolate; DHPS, dihydropteroate synthase; PAS, *para*-aminosalicylic acid; THF, tetrahydrofolate; TYMS, thymidylate synthase.

Instead of simply prioritising individual gene product targets for compound screening, target-agnostic phenotypic approaches yield empirically effective candidates without prejudice towards any particular target. As chemical matter discovered this way progresses through the pre-clinical pipeline, it is advantageous to elucidate mechanisms of action and resultant implications for resistance and drug combinations. Molecular genetics can address this need, and help fill remaining knowledge gaps in the field of antibacterial drug discovery (Figure 2A). For example, the emerging discipline of chemical genomics might enable efficient target dereplication to prioritise chemical matter which could circumvent prevalent resistance mechanisms. Using genetics to build better understanding of how cells modify or exclude chemical compounds would enable prioritisation or design of compounds which are not easily inactivated or excluded. Additionally, once the target of an antibacterial candidate discovered using target-agnostic methods is known, the network of gene–gene interactions would indicate productive combination regimens that increase efficacy and lower the incidence of resistance (Figure 2B).

However, even with the most detailed understanding of pathogen biology, new antibacterial drugs will not be discovered without tools to systematically understand how this biology reacts to perturbation by new natural products and synthetic chemical agents. Similarly, new drugs will not enter the clinic without antibacterial medicinal chemistry for permeability, low toxicity, and pharmacokinetic and pharmacodynamic properties (Figure 2A). Therefore, the next phase of antibacterial drug discovery will be enabled by integrating this chemical expertise with systematic dissection of bacterial infection biology.

## Genetics across scales: informing antibacterial drug development

The sustainable efficacy of an antibacterial drug depends on how its effect on gene product activity changes infection outcome, and how likely it is that resistance will evolve either to its mechanism of action or to its chemistry. Genetic perturbations can roughly simulate the effect of a non-competitive drug, but their pertinence in drug discovery is in understanding genes’ roles in broader cellular, evolutionary, and chemical biology of infection. Within phenotypic screening paradigms, molecular genetics can also aid target deconvolution for screening hit compounds, enabling subsequent prioritisation on target attractiveness. Multi-scale molecular genetics can assess several factors influencing this attractiveness, including dominance of its loss of function, dependence of this dominance on genomic context, kinetics of phenotype, and its mutability (Figure 2A).

### Genes: loss-of-function dominance

**Figure 3.**
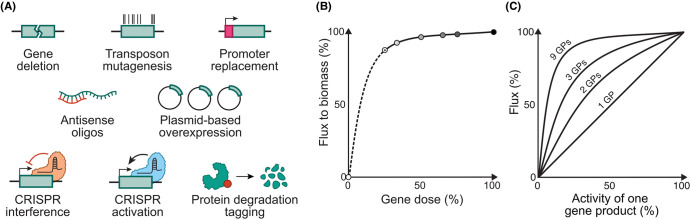
Genetic tools to understand the function of specific drug targets as well as their context in wider biological networks. (**A**) Schematic of methods to determine gene essentiality, function, and loss-of-function dominance. (**B**) Growth of *S. cerevisiae* mutants at the *ad-2* locus, involved in purine biosynthesis. Black circle indicates wild-type activity, grey circles indicate triploid and tetraploid strains with null and wild-type allele combinations. (**C**) Effect on flux of decreasing activity of one gene product (GP), with increasing pathway size (1, 2, 3, and 9 gene-products). Adapted from Kacser and Burns [25].

Conventional antibacterial drugs inhibit one or more gene products whose function is essential. Since the concentration of drugs in a host's tissues fluctuate between doses, ideal targets are those upon which a bacterial phenotype, such as growth rate, is exquisitely dependent, so that modest loss of function causes catastrophic loss of fitness. Recent genetic tools provide a means to assess thousands of genes in parallel for this loss-of-function dominance (Figure 3A).

Gene deletion has for decades been used to understand fitness dependence on gene dosage in model polyploid species like *Saccharomyces cerevisiae*, but this method cannot be trivially extended to essential genes in haploid bacteria, since deletion is lethal [25]. Generating clean gene deletion mutants quickly becomes laborious at the genome scale, but their utility drove investment in deletion libraries of all non-essential genes for model organisms (*Escherichia coli*, *Bacillus subtilis*) and some pathogens (*Acinetobacter*, *Salmonella* spp.) [26–29]. Unfortunately, such libraries do not exist for many important pathogens including *Klebsiella pneumoniae*, *Pseudomonas aeruginosa*, and *M. tuberculosis* because genetic manipulation in these species is inefficient or only recently implemented [30,31]. The effect of gene deletion on fitness can also be defined through disruption of open reading frames by random transposon insertion, usually resulting in inactivation of the target, and has been used to create single mutant libraries in less genetically tractable species such as *P. aeruginosa*, *K. pneumoniae*, *M. tuberculosis*, *Neisseria meningitidis*, *Vibrio cholerae*, and *Francisella novicida* [32–43]. Comparison of essentiality analysis from deletion and transposon insertion libraries showed overlap between each method, but transposon libraries revealed greater resolution on essential regions of genes, polarity effects, and other genomic features like protein secondary structure. For example, a single insertion in the essential region of nucleotide exchange factor *grpE* demarcates a flexible region between two essential α-helices involved in DnaK binding [44,45]. Like other high-throughput methods, transposon-based methods are limited by the polar effect arising from disrupting operon structure, sequence insertion bias, insufficient saturation, and *trans*-complementation during pooled growth [45–47].

Given that essential genes tend to be highly conserved, antibacterial drugs targeting essential genes often possess broad-spectrum activity [48]. However, the dichotomy of essentiality does not capture the continuous response of phenotype to gene dosage. For haploid organisms (including most bacterial pathogens), this continuous response can now be measured using more recent molecular techniques interfering with transcription, translation, and post-translational processes.

Ideal inhibitors would target an essential gene whose modest loss-of-function would result in a large fitness decrease. Loss-of-function dominance (also termed vulnerability), is the quantitative relationship between gene-product activity and organism fitness, first studied by reducing gene dosage in polyploid organisms (Figure 3B,C). In principle, vulnerable genes make good drug targets because small molecules might not always realise total inhibition of gene product activity, and invulnerable essential genes can tolerate such partial inhibition without fitness impacts [49,50]. Additionally, invulnerability implies that most mutant alleles are recessive in fitness, allowing exploration of genetic space — including evolution of target-based drug resistance — without catastrophic fitness loss. After target deconvolution for hits from phenotypic screening, an important property to inform prioritisation of candidates is the dominance of their target's loss of function.

To that end, measurement of phenotypes as a function of gene transcription levels was enabled for the first time by controlling the transcription of single genes of interest through expression from a plasmid or fusion of synthetic promoters to the endogenous open reading frame [51–57]. Enabling genome-scale application of this approach, CRISPR interference (CRISPRi) sterically blocks native transcription at a programmed locus using a catalytically inactive Cas9 nuclease (dCas9) directed by a guide RNA (gRNA) protospacer provided in *trans* [58,59]. The extent of CRISPRi can be modulated through titration of dCas9 or gRNA expression, mismatches between the protospacer and target sequence, or protospacer adjacent motif sequences [50,60,61]. Although mitigation strategies are emerging, CRISPRi can, like transposon mutagenesis, exert unintended polar effects on polycistronic operons and off-target loci [62–68]. Despite these drawbacks, CRISPRi has been applied extensively in *E. coli*, *B. subtilis*, and *M. tuberculosis*, where pooled and arrayed libraries targeting each gene or tiled across the genome have been used to survey vulnerability and function, including comparison between strains and conditions [63,68–72]. Measuring phenotypes beyond simple fitness, screens have combined CRISPRi with imaging, animal infection models, and study of gene–environment interactions [70,73–75].

CRISPR activation, where CRISPRi machinery is paired with a transcriptional activator to recruit RNA polymerase and up-regulate transcription, is well-established in eukaryotes but equivalent prokaryotic tools are still under development [58,76–80]. Refinement of this technique will allow investigation of targets amenable to non-inhibitory chemical modulation, currently only achievable through lower-throughput promoter replacement tools, such as the DegP and ClpP proteases which are activated by antibiotic acyldepsipeptides (ADEPs) [81]. However, transcriptional modulation only provides a partial model of chemical interaction with gene products, and can be confounded by translation rate and protein stability [82].

Translation can be manipulated using antisense oligos to bind a gene transcript resulting in steric hindrance of translation or degradation of the antisense-RNA complex [83,84]. Shotgun antisense RNA (asRNA) cloning enabled comprehensive genome-wide screening, but identified essential genes can be variable: two separate asRNA assays in *Staphylococcus aureus* identified 150 and 658 essential genes, and an asRNA screen in *E. coli* only identified one-quarter of essential genes previously identified by gene deletion [26,85–88]. This discrepancy may result from incomplete asRNA silencing or leaky asRNA expression preventing generation of knockdown strains targeting essential genes expressed at a low level. Nevertheless, antisense oligos are well-suited to study of riboswitches as drug targets, and have themselves been developed as antibacterial agents [89,90]. However, conventional antibacterial drugs act post-translationally; therefore, genetic tools acting similarly give important insights into the biology of drug targets.

The first techniques for tuneable post-translational proteolysis applied carboxy-terminal SsrA-like tags to a coding sequence of interest and used adaptor SsrB to regulate degradation of the tagged fusion by caseinolytic protease Clp [91–93]. Later developments involved chemically induced reassembly of split adaptors, regulation through tag cleavage, or orthogonal tags and heterologous machinery [94–96], while orthologous methods for protein degradation include BacPROTACs [97]. Protein degradation is a fast-onset perturbation and enables investigation of long-lived proteins whose copy number is robust to transcriptional modulation. However, it does not recapitulate conventional chemical inhibition, where the protein remains intact and can perform other functions such as scaffolding, alternative reactions, or substrate wasting. For example, fluoroquinolones cause DNA gyrase to generate breaks in DNA, which does not happen on transcriptional or post-translational depletion of the enzyme [98,99]. Additionally, not all targets are susceptible to proteolysis, and, as shown in studies on *E. coli* and *M. tuberculosis*, not all genes are amenable to tagging, potentially due to interference with functionally essential multimerisation [96,100].

Importantly, gene dose–phenotype response is distinct from any compound dose–response, partly because the response curve will depend on whether a compound acts competitively or allosterically, partly because most chemical inhibitors act post-translationally (thus subject to different compensatory mechanisms, such as metabolite feedback inhibition), and partly because compound affinity and partitioning will determine the degree of gene product inhibition and thus its phenotypic consequences. A genetically vulnerable gene whose product is undruggable is chemically invulnerable, whereas a genetically less vulnerable gene for which a high affinity inhibitor exists is chemically vulnerable. This concept is demonstrated by the handful of bacterial genes that have known chemical inhibitors, compared with the hundreds of vulnerable genes that have been reported. Effective drugs are more than their targets; therefore, integrated biological and chemical approaches are indispensable for developing new antibacterial drugs.

Nevertheless, loss-of-function dominance is an attractive property of potential drug targets, emergent from a gene product's kinetic linkage to other genes through shared metabolites and protein–protein interactions. Loss-of-function dominance is, therefore, dependent on the gene product's context within metabolic and regulatory networks [25], resulting in a non-linear relationship between gene product activity and fitness (Figure 3B). Because vulnerability depends on local interactions in biological networks, systematic identification of gene–gene interactions would deepen our understanding of vulnerability as well as its contribution to the evolution of resistance (Figure 3C).

### Interactions: metabolic control and synergy

To quantitatively elucidate the kinetic linkage between two gene products, they must be modulated in combination [101]. In *S. cerevisiae*, systematic study of genetic interactions yielded information on functional and regulatory relationships, enabling functional prediction for uncharacterised genes [102,103]. Quantifying genome-wide epistasis in bacteria will reveal genes that are metabolically coupled and therefore constrained to co-adapt. This knowledge could lead to prediction of available evolutionary routes in response to antimicrobials. Additionally, genetic interactions can indicate synthetic lethality to be exploited or synthetic rescue to be avoided in drug combinations.

In haploid bacteria, although double deletion mutants can be constructed to measure a single interaction between two genes, this approach is limited to pairs of non-essential genes, and yields only binary rather than quantitative data on gene–gene interactions. Current low-throughput methods involve bacterial conjugation, which is hampered by false positive interactions arising from decreased recombination efficiency between genes within 60 kb of each other, and arrayed plate growth, limiting throughput compared with pooled methods [104,105]. Alternatively, transposon insertion sequencing can be performed in a mutant background, but this has not yet been applied to all gene–gene pairs [32].

Barcoded shotgun and recombineering strategies for overexpression has dramatically increased throughput, affording pairwise and higher-order combinatorial overexpression of transcription factors to identify network interactions that potentiate antibiotics [106–109]. CRISPRi made gene–gene titration accessible through combination of CRISPRi libraries and gene deletion collections, or use of multiple co-ordinately controlled gRNAs to quantify synergistic and synthetically lethal interactions [59,110–114]*.* Recently, mismatch CRISPRi has been used in *E. coli* to generate independently titrated pairwise gene knockdowns for 19 genes, but genome-wide quantification of genetic epistasis represents a combinatorial challenge that has not yet been resolved for any organism [115].

The fitness of mutants in the presence of a chemical compound, i.e. chemical–genetic interactions, can also be used to evaluate antibacterial drug targets [98]. In diploid organisms, heterozygous gene deletion produces strains hypersensitised to drugs that act on the corresponding gene product. Therefore, the combination of gene deletion and chemical screening allows identification of compound mechanisms of action for target dereplication [116]. Combination of deletion and transposon insertion libraries with antibiotics and other chemical stressors elucidated non-essential gene function, conditional essentiality, and chemical–genetic interactions, whilst also identifying antibiotic resistance determinants and secondary targets whose inhibition could potentiate existing antibiotics in combination therapy [117–125]. For example, hypersensitive asRNA knockdown strains were used in natural product screens to identify inhibitors of fatty acid synthesis and ribosomal machinery [126–128]. Similarly, the PROSPECT assay combines sensitised strains produced through degradation tagging with chemical libraries, enabling systematic screening for new compounds with whole-cell activity [129].

CRISPRi can also be combined with chemical screening, allowing prediction of chemical mechanisms of action and synergistic targets, and identification of intrinsic drug resistance mechanisms [71,130,131]. Metabolic profiling of CRISPRi and chemical perturbations identified shared signatures between chemical inhibition and target knockdown. However, some targets present exceptions where genetic knockdown does not phenocopy or causes opposite effects to antibiotic action, such as the effect of fluoroquinolones on *gyrA* [130], further emphasising the risks of overinterpreting transcriptional inhibition as chemical modulation.

Chemical–genetic interactions can also reveal resistance liabilities of antibacterial drugs. For example, in the PROSPECT study [129], the MshC knockdown strain of *M. tuberculosis* was resistant to isoniazid, highlighting mycothiol biosynthesis loss of function as a resistance pathway [132]. The same work showed the ThyA (thymidylate synthase) knockdown to be resistant to folate biosynthesis inhibitors, a phenomenon recapitulated in the clinic with *thyA* deletion conferring resistance to *para*-aminosalicylic acid, a dihydropteroate synthase inhibitor (Figure 2B) [133]. However, in evaluating resistance liabilities of antibacterial drug targets, disruption of entire gene products can only model resistance conferred by loss of function, whereas prevalent examples of resistance are conferred by acquisition of new alleles, or abolition of target binding through mutation.

### Mutability: acquisition of resistance

The driver of our constant search for new antibacterial drugs is resistance, which emerges from interactions between a drug's chemistry and multi-scale pathogen biology from genes through communities and host–pathogen interactions. Genes encoding resistance to β-lactams, tetracyclines, and glycopeptides have been identified in environmental samples dating back 30 000 years, and phylogenetic analyses indicate antibiotic synthesis and resistance genes coexisted hundreds of millions of years ago [134–137]. Evolution of antibiotic synthesis genes and resistance mechanisms was likely driven by early competition [138–141]. Such resistance mechanisms can be generic (reduced influx, efflux), target specific (target modification, variation, or protection, metabolic bypass), or class-specific (compound modification, prodrug activation, efflux), and mobilised between species though horizontal gene transfer. Since many different forms of resistance to natural product antibiotics are widespread in non-pathogenic environmental bacteria [142,143], it is increasingly important to proactively assess resistance liabilities of both the chemical class and the target of antibiotics in development.

Whereas resistance to natural products commonly occurs through gain of function resistance transmitted by mobile genetic elements, including target protection and drug inactivation, it can also occur through target mutation or even polymorphisms that are apparently unrelated to the mechanism. For example, mutation of RNA polymerase subunits in *Neisseria gonorrhoeae* confer resistance to cefalosporins which inhibit cell wall biosynthesis [144]. Similarly, resistance to synthetic antibiotics often occurs through chromosomal mutation of their target or prodrug activating enzymes [145–147]. Therefore, when prioritising new small molecules for their potential as antibacterial drugs, it is important to consider the resistance liabilities conferred by both their chemistry and their targets. New resistance-conferring alleles, such as inactivating enzymes [148] or efflux pumps [149], usually act on the chemistry of a drug and originate as immunity factors or mutated biosynthesis genes in the producer of a given natural product antibiotic [150]. Alternatively, target mutations reduce the binding of a compound while retaining essential target function, as with rifampicin, which inhibits RNA polymerase. For these cases, molecular genetics enables quantification of target mutability and plasticity of compensatory pathways which would allow regimen design to anticipate resistance.

The propensity for a target to acquire resistance while retaining enough essential activity to maintain viability depends on three influences. First, evolution has tuned genomic stability by balancing the opposing forces of DNA repair fidelity and DNA damage, both by endogenous mechanisms (such as replication–transcription conflicts [151]) and by exogenous insults (such as host reactive oxygen species, ultraviolet light, and drugs like mitomycin). These forces depend on local nucleotide sequence, structure, and gene expression; therefore, a target's mutability depends partly on its encoding gene's accessibility to mutagenic mechanisms. Second, the dominance of the gene's loss of function determines the tipping point of the resistance-fitness trade-off. Third, the functional fragility of a target will determine which regions of genetic sequence space are under purifying selection. If a protein target's activity or function can tolerate few mutations, then it is less likely that one of those mutations would confer resistance to a drug. For example, the small molecule BRD-8000.3 inhibits *M. tuberculosis* EfpA, an essential efflux pump, but the handful of target-based resistance mutations also cause severe fitness defects, and multiple mutations are empirically not tolerated [152].

The fragility of gene function is also influenced by context within metabolic, regulatory, and protein–protein interaction networks, as demonstrated by the influence of a prevalent carbonic anhydratase mutant of *N. gonorrhoeae*, which causes CO_2_-dependence but compensates fitness loss from DNA gyrase mutations, which confer fluoroquinolone resistance [153]. On the one hand, biological networks define evolutionary constraints with respect to metabolically coupled genes (e.g. dihydrofolate reductase and thymidylate synthase), directly interacting gene products (e.g. two-component signalling systems), or functionally gratuitous dimers with hydrophobic interfaces (e.g. steroid hormone receptors in eukaryotes) [154–156]. These constraints ensure that core metabolism is conserved across the domains of life because it is limited by thermodynamics, stoichiometry, and requirements for certain reaction products [157]. On the other hand, network complexity ensures that no single gene product is responsible for a rate-limiting step, but rather all gene products exert differing degrees of metabolic control on a pathway. This metabolic control emerges from the kinetics of molecular interactions, which also determine the clinical efficacy of antibiotic drugs.

### Time: phenotypic kinetics

Typical serum concentrations of a drug during antibacterial chemotherapy display a pulsatile time-dependence; therefore, an ideal drug target will be vulnerable to lower drug concentrations and have long-lasting phenotypic effects even after brief treatment. The post-antibiotic effect, where bacterial growth is suppressed after removal of drug, depends on both antibiotic concentration and exposure time, with this lag being attributed either to dissociation of antibiotic from the target (a chemical property) or to resynthesis of the target (a biological property) [158–161]. Similarly, the duration of chemical stress dictates the bacterial cell response to such stress [162]. Clearly, duration of drug exposure is a key consideration when defining therapeutic dosage and schedule. It is especially important for pathogens that occupy niches difficult for antibiotics to penetrate, such as lesions formed during *M. tuberculosis* infection [163].

To explore the sensitivity of targets to inhibition duration, one effort measured depletion sensitivity, the speed of growth inhibition after gene inactivation through transposon mutagenesis [164]. The resulting metric was used to rank essential processes by their importance for replication. Due to the inherent differences between chemical inhibition and genetic inhibition, temporal vulnerability to an inhibitor cannot be interpreted from depletion sensitivity, because depletion sensitivity will be affected by transcript dose, protein levels and turnover, which are unlikely to factor into chemical inhibition of a gene product in the same way.

So far, phenotypic kinetics has only been studied in single representative strains from a given species. However, since many bacterial species have large open pangenomes and high variation in gene content within a species, strain background will influence most molecular genetic characterisations relating to the targets of antibacterial drugs [165].

### Genomic context: spectrum of activity

Within *Enterobacteriaceae*, wide variation in essentiality has been identified between strains of the same species, and up to one-third of essential genes are non-essential in other species within the same genera [63,166]. Essential genes have recently been reclassified to describe this flexibility: universal (present and essential across the pangenome), core strain-specific (present in all strains, essential in some), and accessory essential (essential when present) [167]. Importantly, core strain-specific and accessory essential genes can become non-essential with genetic background changes.

This effect propagates to chemical inhibition. Studies of MetS inhibitors in *Streptococcus pneumoniae* demonstrated highly heterogenous activity between strains, with 20% of strains resistant to the inhibitor due to a second redundant MetS that is widespread but not universal [168]. Therefore, genomic context is critically important when examining the properties of antibacterial drug targets, but recent work indicated that this issue is more tractable than might be thought, since analysis of only four strains was sufficient to identify core essential genes shared across strains and relevant growth conditions [45]. Nevertheless, this threshold will likely differ by species, and even single polymorphisms can affect secondary phenotypes like the ability to evolve drug resistance [153]. Transposon mutant libraries can easily be made in multiple strains of a species (including clinical isolates) to allow understanding of variation in essentiality across the pangenome, but they can also be easily subjected to a range of environments, including infection [45,169,170]. If not studied under relevant conditions, empirically high value targets identified by such screens can be misleading.

### Conditions: environmental and host interactions

Cautionary tales abound of antibacterial candidates targeting gene products which are essential in axenic culture but dispensable during infection. In one example, pyrimidine–imidazole inhibitors were developed against for *M. tuberculosis* with high *in vitro* potency but no *in vivo* activity because the target, glycerol metabolism, was irrelevant during infection [171–173]. This effect extends to the fundamental study of gene essentiality, where screening of 24 conditions allowed identification of novel condition-specific essential genes in *Staphylococcus epidermidis*, as well as those vital for adaptability to environmental pressures [66]. In addition, a study of essentiality utilising two pathogens in co-infection models identified ∼200 community-dependent essential genes with altered essentiality compared with monoculture [174]. Infection is inherently heterogeneous; therefore, the properties of antibacterial drug targets must be characterised under multiple conditions which recapitulate fundamental infection processes.

## Chemical biology: bridging the gap between genetics and treatment

A sustainable supply of antibacterial drugs requires the integration of chemistry with biology to uncover new sources of bioactive compounds, to manipulate scaffolds to evade resistance mechanisms like efflux and enzymatic inactivation, and to rationally compose regimens which anticipate and suppress resistance. Historically, and very successfully, natural products were extracted from environmental isolates and simply tested for their ability to kill bacteria, but in the regime of diminishing returns we have encountered a bottleneck in natural product discovery. Now, emerging platforms provide purchasable, ultra-large, synthetically accessible chemical spaces of tens of billions which are impossible to test exhaustively, even *in silico* [175]. Facing the ambitious endeavour to find new molecules in nature or in the laboratory which are active against pathogenic bacteria, the new capabilities furnished by modern chemical biology and cheminformatics can be brought to bear to develop therapeutics science and prospect for chemical modulators of new targets which circumvent prevalent resistance mechanisms.

### New chemical space: engaging new targets

**Figure 4.**
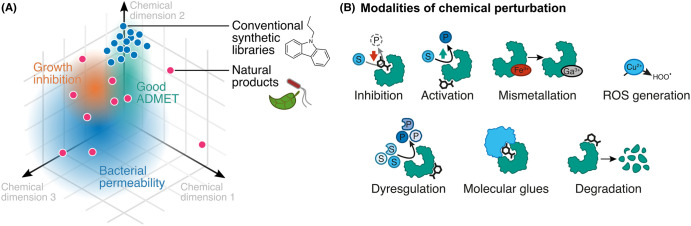
Exploring chemical space for bioactivity and new mechanisms of action. (**A**) Schematic of a region of chemical space including bioactive compounds. The axes indicate dimensions of chemical space, the coloured clouds indicate areas with properties important for antibacterial drugs, and the coloured dots indicate compounds. Only a subset of compounds will permeate bacterial cells, while a minority of these will inhibit bacterial growth while demonstrating good absorption, distribution, metabolism, excretion, and toxicity (ADMET) properties. Conventional combinatorial synthetic libraries (blue dots) cover a narrow chemical space and have been curated for human cell efficacy; therefore, few have found application as antibacterials. Many natural products (magenta dots) cover a wider chemical space and were curated by microbial antagonism for antibacterial efficacy. Synthetic libraries covering wider chemical space could be also artificially curated to prioritise bacterial cell entry, enriching for antibacterial activity. (**B**) An individual target could be modulated by a small molecule agent in a variety of ways, depending on the agent's specific structure.

Recent approaches to natural product prospecting demonstrate a widening of the discovery bottleneck. Making culturable the once-unculturable soil microbes has yielded promising new antibacterial candidates — including teixobactin [176], clovibactin [177], and darobactin [178] — within 9 years, demonstrating that sampling a fresh region of chemical space can readily yield new antibacterial compounds (Figure 4A).

To explore natural product-inspired chemistry, and to circumvent low cultivability of producers or low expression of native biosynthetic pathways which hamper natural product discovery [179], synthetic biologists first engineered existing natural product biosynthesis pathways in 1985, isolating new compounds mederrhodin and dihydrogranatirhodin from strains carrying combinations of the actinorhodin, granaticin, and medermycin biosynthetic pathways [180]. Over the last 40 years, revolutions in molecular biology and computational techniques allowed identification of new biosynthetic enzymes and engineering of known enzymes, manipulation of assembly-line order and composition, diversification of substrates, and optimisation of heterologous host strains [181–183]. However, re-discovery remains a problem despite synthetic biology and combinatorial chemistry approaches to modify natural products; therefore, the potential for pre-existing environmental resistance remains [15,178,183–189].

But untapped regions of chemical space need not necessarily be natural products, as testified by antituberculosis approvals and recent studies screening synthetic cyclic peptides, which readily yielded new clinical candidates like zosurabalpin (RG6006, in Phase I trials) [190]. Peptides and their derivatives represent a further constrained chemical space which can be explored densely if not exhaustively, as exemplified by recent discovery of complestatin, representing a new glycopeptide class with a novel mechanism of action. Another strategy seeks increased antibacterial potency through incorporation of metal-binding motifs into existing antimicrobial peptides such as anoplin [190–192], where the membrane-disrupting action was potentiated by the generation of reactive oxygen species by complexed copper, resulting in membrane lipid damage [193]. This mechanism is reminiscent of the natural product antibiotic and anticancer agent bleomycin, which binds iron and nucleic acids to cleave DNA and RNA through reactive oxygen species generation, but is too toxic to be used as an antibacterial drug. However, with a better understanding of the medicinal chemistry of organometallic antibiotics, the specificity of drugs like bleomycin might be tuned to selectively kill bacteria [194], perhaps by engineering selective permeation of bacterial cells.

### Bacterial permeability and ADMET: reaching new targets

The subcellular location — cytoplasm, periplasm, or within the outer membrane — of a target also plays a role in its attractiveness. While Gram-positive bacteria possess a single membrane and a thick peptidoglycan layer, Gram-negative bacteria possess two membranes, separated by the periplasm and a thin peptidoglycan layer that must be traversed by a compound to reach a cytoplasmic target [195]. In general, targets on the outer membrane or in the periplasm, such as glycopeptides and penicillin-binding proteins, are more easily accessible [191,196–198]. Illustrating this phenomenon, accessible targets, like MmpL3 and DprE1, are over-represented from whole-cell *M. tuberculosis* screening studies due to their localisation on the membrane and the tendency towards hydrophobicity of screening libraries [199]. Some target redundancy accounts for attrition in the pre-clinical pipeline and can effect multiple mechanisms of action through the same target [152], but target diversity is also desirable although out of reach without sampling new regions of chemical space. Libraries designed with permeability in mind would expand the definition of chemically vulnerable targets.

To this end, studies measuring compound accumulation in bacterial cells attempted to define the chemical properties required for bacterial cell uptake. While these properties vary according to envelope structure and mechanism of entry, understanding of some of them has enabled modification of existing Gram-positive antibiotics into compounds potent against Gram-negative bacteria [200–209]. This work also highlighted that even within Gram-negative bacteria and among strains of the same species there are large differences in the cell envelope, making cell entry rules species-specific [210,211]. While the resulting rules were applied to dialling-in Gram-negative activity of fabimycin [212], a proof-of-principle screen of a compound library curated from proprietary collections using these rules did not appear to yield any hits which could be progressed [213], indicating that there is probably still more to understand about bacterial cell entry by small molecules. An alternative approach to permeability is the Trojan horse strategy, originating with natural product antibiotic-siderophore conjugates like albomycin that chelate iron and are taken up by bacterial iron transporters to generate high intracellular antibiotic concentrations [214,215]. This approach has seen clinical approval with cefiderocol, a cephalosporin-catechol conjugate, and has also been used to sensitise the Gram-negative *Acinetobacter baumanii* to the Gram-positive antibiotic daptomycin by conjugation to a siderophore mimic [216,217].

However, permeation into the bacterial cell is only the first step towards clinical efficacy of a small molecule. For example, during tuberculosis infection, granulomas can shelter *M. tuberculosis* from isoniazid [218], while the partitioning of bedaquiline into lipid droplets in macrophages boosts its antituberculosis activity [219]. Antibacterial drugs are also subject to similar absorption, distribution, metabolism, excretion, and toxicity (ADMET) requirements as other therapeutics, with some additional considerations on toxicity and route of administration. Antibacterial drugs are usually taken at high doses over the course of a few weeks, except for the treatment of diseases caused by mycobacteria, such as tuberculosis (6–18 months) and leprosy (6–12 months), and prophylaxis for immunocompromised patients, which uses lower, long-term dosing. In the present paradigm of ‘use it and lose it', new antibacterial drugs will typically be used as a last resort, so that some toxicity is tolerated by regulators. For example, the use of colistin, which is nephrotoxic, was abandoned in the late 20th century, but its use was revived in the early 21st century as a drug of last resort [220]. However, in low-resource settings and for diseases with long-term treatments, supportive care to complement toxic therapies may not be accessible, making toxicity is a more pressing consideration. The difference in ADMET requirements between antibacterial drugs and drugs for diseases not caused by bacteria can be illustrated where they intersect, for example with doxorubicin, an intravenous topoisomerase inhibitor which causes immunogenic cell death in cancer at in the low nanomolar range, and which also kills mycobacteria in the low micromolar range [221]. It is barely tolerated as an antineoplastic agent, and probably would never be used in its present form as an antituberculosis drug.

Similarly, desirable routes of administration also vary by disease, the plurality of alternative therapeutics, and availability of clinical resources. For example, intravenous dosing often avoids first-pass metabolism, but it requires expert attention which is not always available in primary care. In contrast, oral dosing requires greater investment in medicinal chemistry optimisation, and, for a given chemical scaffold engaging a promising target, antibacterial activity and oral bioavailability may be mutually exclusive. Therefore, when assembling chemical libraries for phenotypic screening, it is essential to curate scaffolds which are predicted to be or a short derivatisation from being orally bioavailable.

Alongside considering host metabolism, bacterial metabolism can also present opportunities and challenges to medicinal chemistry. Enzymes which modify antibacterial candidates can inactivate them, or in the case of prodrugs, activate them. Such activating modifications can allow an inactive-but-permeable chemical species to reach the bacterial cytoplasm and acquire an active-but-impermeable form which accumulates inside the cell, as readily visualised for the dye calcein acetoxymethyl ester [222], and as is the case for the antituberculosis prodrug isoniazid [223]. Some competitive inhibitors of two-substrate enzymes, such as dihydropteroate synthase inhibitor sulfamethoxazole, also act through a secondary mechanism of substrate wasting where the drug forms an adduct with an essential metabolite, thus depleting it. This mechanism precludes resistance through target amplification, since overexpression only exacerbates this substrate wasting [98]. As demonstrated with *para*-aminosalicylic acid, the drug-metabolite adduct can also inhibit enzymes downstream of the parent drug's target [99], increasing the barrier to resistance.

### Beyond inhibition: effecting new phenotypes through chemistry

The ability to bind small molecules to a target is a basic yet important requirement for target selection [224]. Many antibiotics, like penicillin, achieve target inhibition by binding to an enzyme's active site, but others bind elsewhere on their target protein, like rifampicin which binds to the DNA/RNA channel of RNA polymerase to block the path of elongating RNA [225,226]. Compounds with non-traditional modalities expand our ability to modulate targets beyond simple inhibition, such as the activation and dysregulation of ClpP by ADEP and ACP compounds, or over-activation of DegP by tripodal peptidyl compounds (Figure 4B) [227–229]. Pyrazinoic acid, the active form of the first-line tuberculosis antibiotic pyrazinamide, has been shown to trigger degradation of PanD rather than traditional functional inhibition [24]. The recent development of BacPROTACs offers a generalisation of this strategy, since they reprogramme the ClpCP system in Gram-positive bacteria to achieve specific degradation of proteins [97].

Metal-based compounds offer a range of alternative mechanisms of action, for example, mismetallation of iron-dependent enzymes by gallium complexes results in growth inhibition and biofilm disruption (Figure 4B) [192,193,230,231]. Molecular glues cause non-natural interactions between biomolecules, but none have been discovered for bacterial pathogens, despite the use of thalidomide — a cereblon ligand often used to design proteolysis targeting chimaeras in human cells — since the 1960s as a host-directed therapy to treat complications of leprosy, caused by *Mycobacterium leprae* [232].

Genetically encoded targets are not the only possible targets within a cell, demonstrated by glycopeptides such as vancomycin, ramoplanin, and complestatin which bind cell wall components to prevent cross-linking, transglycosylation, and cell wall remodelling [191,233,234]. Non-encoded targets such as lipids and glycans represent an underutilised class; therefore, new techniques are needed to evaluate them as antibacterial drug targets. Chemical probes to study and modulate both genetically encoded and non-encoded targets will prove invaluable, such as use of trehalose analogues to study pathway-dependent labelling of mycobacterial glycolipids, or study of post-translational modification through bump-and-hole engineering, recently used to identify substrate specificities of particular isoenzymes *in vivo* through engineered glycosyltransferases and tagged glycans [235–237].

## Unlocking the third wave of antibacterial drug discovery

To sustain the repertoire of antibacterial drugs with clinical utility, discovery of non-toxic, potent small molecules with new mechanisms of action must outpace emergence of new resistance. This challenge can be met with the integration between chemistry and genetics, as highlighted by recent efforts in exploring the antibacterial chemical space, deriving resistance-proof natural products, and anticipating new resistance mechanisms.

### Prioritising unexplored chemical space for likely bioactivity

The ADMET criteria for drugs to treat bacterial infections are distinct from those to treat diseases like cancer and diabetes. Since the vast chemical libraries of large pharmaceutical companies are biased towards the ADMET and cell permeability properties for activity in human cells, it is possible that the apparent failure of target-based antibacterial drug discovery — where compounds active *in vitro* failed to penetrate the bacterial cell wall — was caused more by unsuitable chemical matter than by flawed methodology. Decades of research on human diseases in general has defined heuristics to guide curation of suitable chemical libraries [238–240], but there are no such comprehensive guides for antibacterial drugs, and worse, some properties which are broadly considered unfavourable are the definition of success for some of the best antibacterial drugs. For example, nitrofurantoin is both reactive and possesses an aromatic nitro group, properties which would trigger structural alerts and exclude it from most typical chemical libraries but which also drive its efficacy.

The heuristics for curating antibacterial drug discovery libraries will have some overlap with the current rules. Therapeutic index, the gap between therapeutic and toxic doses, must be more conservative, whereas oral bioavailability could be dispensable in the most dire clinical cases. To unlock the intracellular majority of essential targets, chemical libraries optimised for phenotypic screening must be enriched for compounds which penetrate the bacterial cell wall, while those for target-based discovery should contain compounds within a few robust synthetic steps from bacterial permeability. To curate such libraries, reliable models of ADMET properties and bacterial permeability are necessary.

While favourable ADMET properties and bacterial permeability are necessary for antibacterial drugs, they are not sufficient. Unlike other diseases caused by one or a handful of gene products, any essential gene product or other non-encoded biomolecule in bacterial pathogens is a vulnerability, collectively presenting a large surface area for targeting. Whereas antibacterial drug discovery has historically focussed on natural products and therefore new discovery has been stymied by the coupon collector problem, recent works which sampled underexplored chemical space have readily discovered bioactive compounds.

**Figure 5.**
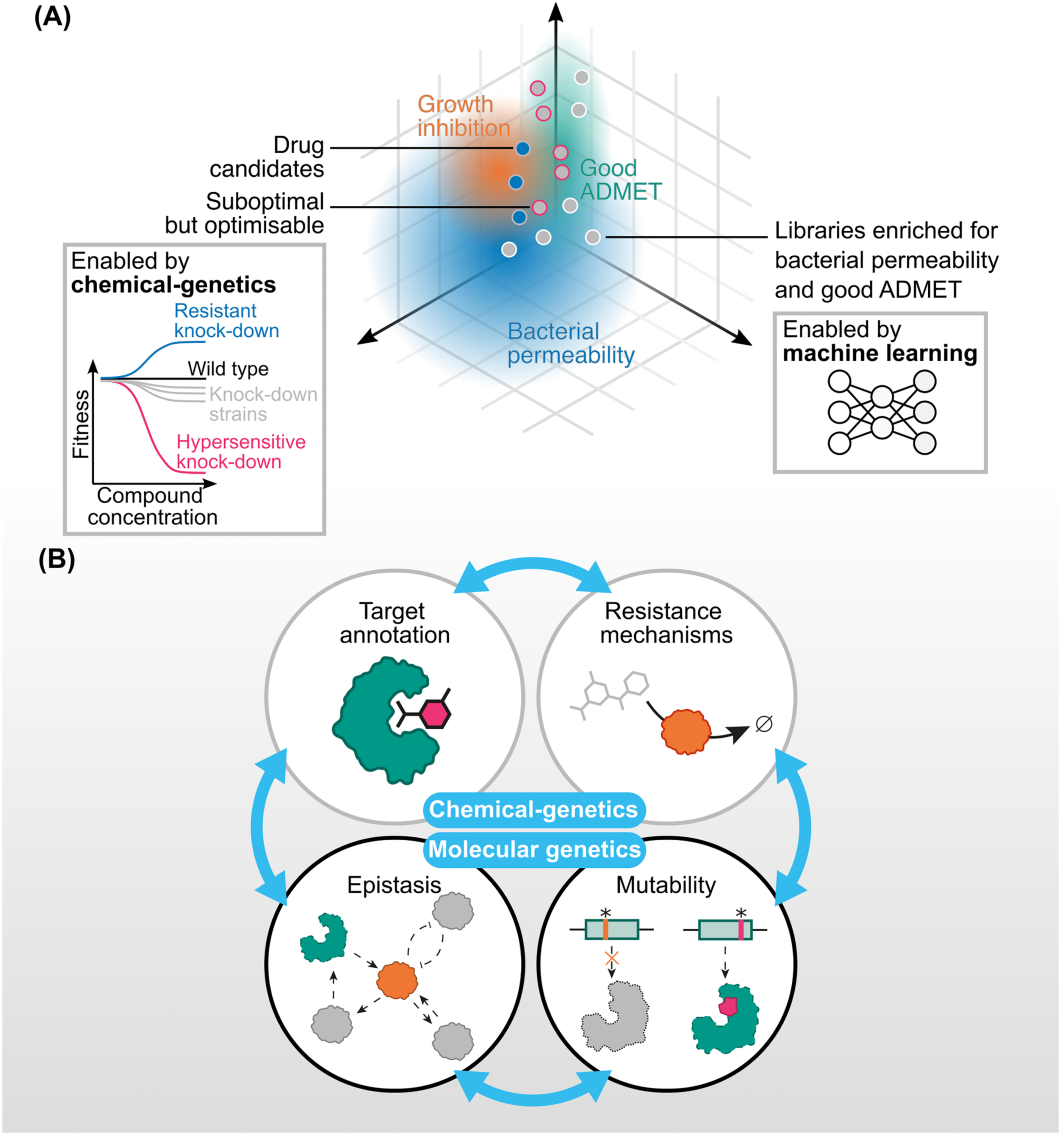
Strategies to unlock the third wave of antibacterial therapies. (**A**) Schematic of screening chemical libraries enriched for bacterial permeability and good ADMET. With appropriate predictors of bacterial permeability and ADMET, potentially furnished by machine learning models, new chemical libraries could be designed with these properties in mind. Using chemical–genetic assays like PROSPECT allow sensitive detection of compounds that are not active against wild-type bacteria but do affect knockdown strains, enabling a sparser search of chemical space and therefore accelerating the identification of new bioactive chemical scaffolds. These scaffolds can then be optimised for wild-type activity. (**B**) Chemical–genetics and molecular genetics provide complementary tools for anticipating resistance and for designing combination regimens which suppress or exploit it.

Defining a new but constrained chemical space to a region expected to be enriched in bioactivity enables systematic, thorough exploration (Figure 5A). For example, zosurabalpin was discovered through a medium-sized screen of 44 985 synthetic macrocyclic peptides [190], a relatively unexplored chemical class. Similarly, new sources of natural products can also yield very high hit rates. For example, bioinformatic identification of biosynthetic gene clusters from the human microbiome followed by their heterologous expression in *E. coli* yielded a handful of new antimicrobial peptides from 70 candidates [241].

Sampling new chemical space has also been directed by strategies employing deep learning. In a recent thread of work, a message-passing neural network was trained on high quality data representing the inhibitory activity of a small set of 2335 unique molecules comprising FDA-approved drugs and natural products, of which 120 had inhibitory activity against *E. coli* [242]. Given the small number of positive training examples, the large number of potential mechanisms of action represented by the hundreds of essential genes in *E. coli*, and the combinatorial vastness of potential chemical features beyond the scaffolds of known antibiotics, the model likely learned to recognise some features of *E. coli* permeability. The trained model was used to curate 23 molecules from the ZINC15 database of >100 million purchasable or readily synthesisable compounds, which might not otherwise have been screened against bacterial pathogens. This approach yielded two compounds with appreciable activity against *E. coli*, demonstrating the potential of sampling new chemical space for antibacterial candidates. Later, the same model architecture was trained on a dataset containing 7684 compounds including 480 actives against the priority pathogen *A. baumanii*, ultimately yielding abaucin [243]. More recent work enriched chemical spaces with favourable ADMET by using models to score both human cytotoxicity and antibacterial potency, trained on 512 positives out of 39 312 compounds screened against *S. aureus*, enabling rapid prioritisation of compounds for phenotypic testing and yielding promising new antibacterial candidates [244].

Even with these computational methods, scoring the ever-expanding subset of readily synthesisable compounds, currently ∼50 billion, is resource-intensive, and out of reach of computational docking screens [245], which are currently limited to hundreds of millions of compounds, although methods like V-SYNTHES which operate in lower-dimensionality reagent space [175] provide a means to sift through enormous virtual libraries. Such tools that enable a sparse search of chemical space will be invaluable to prospect for bioactivity, which can then be mined using chemically focused libraries. To this end, active learning is an emerging framework to iteratively screen subsets of vast chemical libraries [246,247]. By training deep learning models equipped with a measure of uncertainty or variance in their predictions, smaller compound libraries can be enriched with compounds predicted to be high activity with low uncertainty. Alternatively, to improve the model's generalisability to compounds dissimilar to the training set, the next library can contain compounds annotated with maximal uncertainty, so that the updated model can learn from their data. In this way, advanced statistical models can steer a data-driven search for new antibacterial activity.

To enable a sparse search of chemical space in the wet laboratory, bacterial mutants with essential genes knocked down can be used as sentinels of suboptimal but optimisable chemical space. Because essential gene knockdown mutants are hypersensitive to inhibitors of pathways related to their knocked down gene product, their growth is inhibited either at lower concentrations or lower potencies. For example, using the PROSPECT assay, it was possible to identify the inhibitor BRD-8000, which had no measurable activity against wild-type *M. tuberculosis* but inhibited the growth of the *efpA* knockdown [129]. The dimethylcyclopropanyl scaffold of BRD-8000 resembled neurotoxic chrysanthemic acid derivatives used as insecticides, but the specific activity of the compound against a mutant encouraged structure–activity relationship studies which revealed that the (S,S)-enantiomer of BRD-8000 was active, in contrast with the insecticidal (R,R)-enantiomer of chrysanthemic acid. Further medicinal chemistry led to an inhibitor with micromolar potency against wild-type *M. tuberculosis*, demonstrating that unusual or even unattractive chemical space can contain chemical modulators of new targets with phenotypic activity. As with all new antibacterial agents, it will be essential to understand the resistance liabilities of these new mechanisms of action.

### Anticipating and exploiting resistance

Resistance to antibacterial drugs is empirically inevitable with present agents, even when resistance appeared unlikely. For example, since the cell wall tripeptide target of tricyclic glycopeptide vancomycin is not encoded, resistance was thought to be vanishingly unlikely. However, although no vancomycin resistance was detected decades after its discovery in 1957, its eventual clinical deployment in the 1970s ushered in high-level resistance in *Enterococcus* 16 years later [248], a similar time-to-resistance to other antibacterial drugs (Figure 1B). The main resistance mechanisms are carried on a transposable element which confers vancomycin sensing and subsequent biosynthesis of cell wall tripeptides with lower vancomycin affinity [249].

Thus, there should be no complacency with new antibacterial agents about the emergence of resistant isolates, even when it does not easily evolve in the laboratory where, conventionally, high-density bacterial cultures are plated on *supra*-inhibitory compound concentrations. While this approach is often successful, it is prone to false negatives, partly because it focusses on spontaneous mutation at a rate higher than ∼10^–9^ within a monoculture (excluding horizontal gene transfer from the environment or host microbiome), partly because drug concentrations at infection sites are not necessarily constant and *supra*-inhibitory, enabling a stepwise evolutionary path through low-level resistance. For example, *E. coli* have demonstrated the ability to acquire mutations that confer tolerance — slowed death in the presence of normally lethal ampicillin concentrations — which in turn bought time for acquisition of high-level resistance alleles [250]. A similar phenomenon was described in *Mycobacterium smegmatis*, where ribosomal mutations with a fitness cost enabled low-level broad-spectrum resistance followed by high-level resistance and compensatory mutations to restore wild-type growth rates [251].

Additionally, mutation rates during infection may be higher than in broth, as demonstrated by higher rate of rifampicin resistance in cultures derived from patients than those from an axenic culture. Random mutations may also be distributed across the genome differently during infection, since an important mechanism of mutagenesis is replication–transcription conflict [151], where actively transcribed genes orientated against the direction of replication cause stalled replisome and DNA damage, leading to mutation. Since the transcriptional activity of bacteria during infection is distinct from that in axenic culture, it is likely that there are spontaneous mutants resulting in resistance not observed *in vitro*.

As a result, resistance to antibacterial drugs is usually a surprise, Fleming's warnings in 1945 notwithstanding. To be better prepared, recent work has applied deep mutational scanning to exhaustively quantify the effects of target mutations on drug resistance and fitness in DHFR in *E. coli* [252] and PncA, the activating enzyme of pyrazinamide, in *M. tuberculosis* [253]. Expanded efforts across genomes of pathogens combined with determination of infection fitness, collateral sensitivity to existing drugs, and interactions with genetic perturbations modelling undiscovered agents would enable anticipating resistance and exploiting it, potentially with designed combination therapies (Figure 5B).

### From agents to regimens: rationally designing optimal combination therapies

The most successful antibacterial drugs, the β-lactams, are multi-target inhibitors to which spontaneous resistance is rare. Instead, resistance to these agents is usually acquired only through mobile genetic elements like plasmids or transposons [146]. Whilst engineering synthetic antibiotics to target multiple sites is challenging, examples under investigation include repurposed antirheumatic agent auranofin and antimycobacterial agent BB2-50F, which inhibits ATP synthase and succinate dehydrogenase [254–258].

Nevertheless, resistance to these inhibitors does occur; therefore, some efforts focus on preserving and renewing the utility of the current antibiotic repertoire, rather than identifying new molecules. Such adjuvants aim to tackle existing resistance or extend the range of activity of a drug [259–261]. Although only β-lactamase inhibitors are approved adjuvants, there are several under investigation with diverse mechanisms: class I adjuvants directly inhibit (e.g. β-lactamase and efflux inhibitors) or bypass resistance (e.g. teichoic acid synthesis inhibitors and membrane-disruptors), whereas class II adjuvants modulate host processes to increase bacterial killing [262–267]. Other adjuvants increase the working spectrum of antibiotics, by sensitising Gram-negative organisms to Gram-positive antibiotics [268,269].

Another method of prospectively supressing resistance is to combine antibacterial drugs. For example, since the 1950s, the tuberculosis standard of care comprises drugs mostly with single targets and *in vitro* resistance frequencies between 10^–9^ and 10^–6^ [12,270], requiring a combination of four drugs to suppress evolution of resistance. The combination strategy also presents the opportunity to exploit synergistic interactions [12,271–273], which have an additional benefit that, whereas some synergies are broadly conserved, others are species-selective, thus generating narrow-spectrum therapies that minimise harm to the host or their microbiome [267,274,275].

Other combinatorial strategies use synthetic lethal interactions, whereby inhibition of two non-essential gene products, sometimes by a single agent, results in bacterial killing. For example, both MurA1 and MurA2, which are genetically redundant and individually non-essential in Gram-positive *S. pneumoniae* and *S. aureus*, are inhibited by fosfomycin and other synthetic agents, ultimately killing the bacteria [276–281]. However, synthetically lethal therapies are vulnerable to resistance to either drug rendering the combination ineffective, as demonstrated by development of resistance to cancer therapies utilising synthetic lethal interactions between BRCA2 mutants and PARP inhibitors or cisplatin through secondary BRCA2 mutation [282,283]. A strategic approach that exploits resistance development couples drugs with collateral sensitivity, whereby evolution of resistance to one drug renders the bacterium hypersensitive to the second [152,284,285]. To design such strategies, it is essential to understand the structure of underlying genetic interaction networks, and the consequent effects of chemical perturbation.

In an example of this combinatorial paradigm, trimethoprim is usually paired with sulfamethoxazole (a combination known as co-trimoxazole), which inhibits dihydropteroate synthase, an enzyme upstream of DHFR in the folic acid biosynthesis pathway. While both drugs are effective alone, they are synergistic in combination. Knowledge of these compounds’ targets and their network biology (Figure 2B) retrospectively explained the efficacy of co-trimoxazole [286]. Continued development of our understanding of antibacterial targets and bacterial physiology will empower future proactive design of optimal regimens.

Understanding of the target of trimethoprim and DHFR's network context has also revealed resistance liabilities for drugs targeting this essential enzyme. For example, loss-of-function mutations in thymidylate synthase render DHFR dispensable and drugs inhibiting it ineffective [133], or up-regulated RibD, a riboflavin biosynthesis protein, can moonlight as DHFR to enable bacterial survival in the presence of *para*-aminosalicylic acid (Figure 2B) [287]. As new small molecules with new mechanisms of action are discovered, understanding their systems chemical biology will be critical to anticipate and exploit resistance.

## Conclusion

Fundamental pathogen biology in relevant infection models leads to the identification and evaluation of putative targets of new antibacterial candidates. There are a range of techniques with unique advantages and disadvantages, often depending on the target under investigation. However, defining targets solely from a biological perspective is not sufficient: it is important to consider whether these targets can be modulated through chemical approaches, what type of modulation can be achieved, and if compounds capable of achieving these modulations can access the subcellular location within which the target resides. Therefore, understanding which areas of chemical space possess these properties will help either reduce the size of chemical screens for target activity, or increase the number of hits which provide starting material to develop lead candidates.

The vastness of this task can be aided by leveraging deep learning to prioritise candidates from ultra-large synthesis-on-demand chemical spaces [288]. Emerging genome-scale genetic techniques such as deep mutational scanning and metagenome complementation will assist medicinal chemists by prospectively identifying resistance liabilities both of target and chemical class. Alongside molecular genetics in the laboratory, it will be critical to consider the population genomics of pathogens and hosts to define the landscape of natural alleles which will influence the assessment of likely activity and resistance of any given antibacterial regimen. In this way, as a companion to new therapeutics, rapid diagnostics which determine the best course of therapy for a given infection will have an important role, enabled by molecular and population genetics. As host-directed strategies develop for bacterial infections, human molecular genetics and chemical biology will aid their clinical implementation.

Ultimately, integrating molecular genetics with chemical biology will allow exploration of target or compound combinations that possess collateral sensitivity, synergy, or combat likely resistance mechanisms to realise the full potential of new and old antibacterial agents. More than a century after discovering the cause of tuberculosis using methylene blue, a new wave of therapies can be inspired by the integrative chemical biology approach of ‘thinking chemically and acting biologically' [289].

## Data Availability

Code and data used to generate Figure 1 are available at GitHub (https://github.com/scbirlab/2024-Parkhill-BiochemJ).

## Open Access

Open access for this article was enabled by the participation of The Francis Crick Institute in an all-inclusive *Read & Publish* agreement with Portland Press and the Biochemical Society under a transformative agreement with JISC.
